# Complete mitochondrial genome and phylogenetic analysis of a Chinese Eurasian lynx (*Lynx lynx*)

**DOI:** 10.1080/23802359.2018.1524280

**Published:** 2018-10-26

**Authors:** Yuhan Wu, Huailiang Xu, Diyan Li, Meng Xie, Jiayun Wu, Anxiang Wen, Qin Wang, Guangxiang Zhu, Qingyong Ni, Mingwang Zhang, Yongfang Yao

**Affiliations:** a College of Life Science, Sichuan Agricultural University, Ya’an, China;; b College of Animal Science and Technology, Sichuan Agricultural University, Chengdu, China

**Keywords:** *Lynx lynx*, mitochondrial genome, phylogenetic analysis

## Abstract

The complete mitochondrial genome of Eurasian lynx (*Lynx lynx*) from China has been described in this study. It has a circular genome of 16,996 bp with a higher A + T content of 58.65%, and the base composition is A: 32.31%, G: 14.29%, T: 26.35%, C: 27.06%. When compared with the reported individual (KR919624) from China, there are 81 variations and 58 base deletion between the two sequences. The phylogenetic analysis indicated our sequence separated clearly from Eurasian lynx individuals in the previous publications. These results could provide more molecular information for the conservation of Eurasian lynx genetic resources.

Eurasian lynx (*Lynx lynx*), a medium-sized carnivore of the family Felidae, has a wide distribution in the world. In China, the distribution of Eurasian lynx stretches from Xinjiang, Qinghai, through the southwest of China down into the northeast of China. Due to over hunting, habitat destruction, and fragmentation, Eurasian lynx is in a very low density, and thus classified as national second class protected animal in China, it is also on the list of Convention on International Trade in Endangered Species of Wild Fauna and Flora (CITES) (Ablimit et al. 1998; Tian et al. 2002; Wang and Xie 2008). Present study situation on genetic diversity of Eurasian lynx is mainly concentrated in Europe (Hellborg et al. 2002; Spong and Hellborg 2002; Schmidt et al. 2011; Cömert et al. 2018). For the Chinese population, only one complete mitochondrial genome (mitogenome) was reported (Ning et al. 2016). Here, we obtained a muscle sample of Chinese Eurasian lynx from the forest public security of Tianquan county (N 30°3′59″, E 102°46′10″), China. The sample was stored at zoology laboratory, Sichuan Agricultural University (Accession: sicau -lz-A232). In this study, the complete mitogenome of Eurasian lynx was sequenced and characterized, and it could provide more molecular information for the conservation of Eurasian lynx genetic resources.

Total genomic DNA was extracted with the Phenol-chloroform method (Sambrook et al. 2001). Using reported Eurasian lynx (KR919624) mitogenome sequence as a reference, we designed twenty pairs of primer and amplified Eurasian lynx mitogenome successfully.

The mitogenome of Eurasian lynx has a circular genome of 16,996 bp (GenBank accession number MH 706704), including 13protein-coding genes (PCGs), 22 transfer RNA (tRNA) genes, 2 ribosomal RNA (rRNA) genes and a control region (D-loop) which was similar to typical mammalian mitochondrial DNA. The start codon of most PCGs is ATG or ATA while ND2 start with ATC. For the stop codon, seven genes ended with TAA, four ended with single T, while ND1 ended with TA, and the cytochrome b gene ended with AGA. The base composition of Eurasian lynx is A: 32.31%, G: 14.29%, T:26.35%, and C: 27.06%.AT and GC contents of mitogenome are 58.65% and 41.35%, respectively. Remarkably, when compared with the reported sequence (KR919624) from China, there are 81 variations and 58 base deletion between the two sequences, sequence similarity is 99%. The D-Loop is the hypervariable region in mitogenome, which contains 27 variations and total 58 base pairs deletion. In detail, sequence similarity is 94.5%, 58 base pairs deletion are separated in 8 sites, the longest deletion is 28 bp, exhibiting a certain degree of disparity.

To further explore the taxonomic status of Eurasian lynx, a Maximum likelihood (ML) phylogenetic tree was reconstructed in MEGA7, using an alignment comprising 11 complete mitogenome sequences from the order Carnivora, and a grey wolf (*Canis lupus*) as an outgroup (Figure 1). On the phylogenetic tree, all sequences from genus Lynx are clustered as a clade, but our Eurasian lynx is distinct from other Eurasian lynxes clearly, implying a different maternal origin.

**Figure 1.**
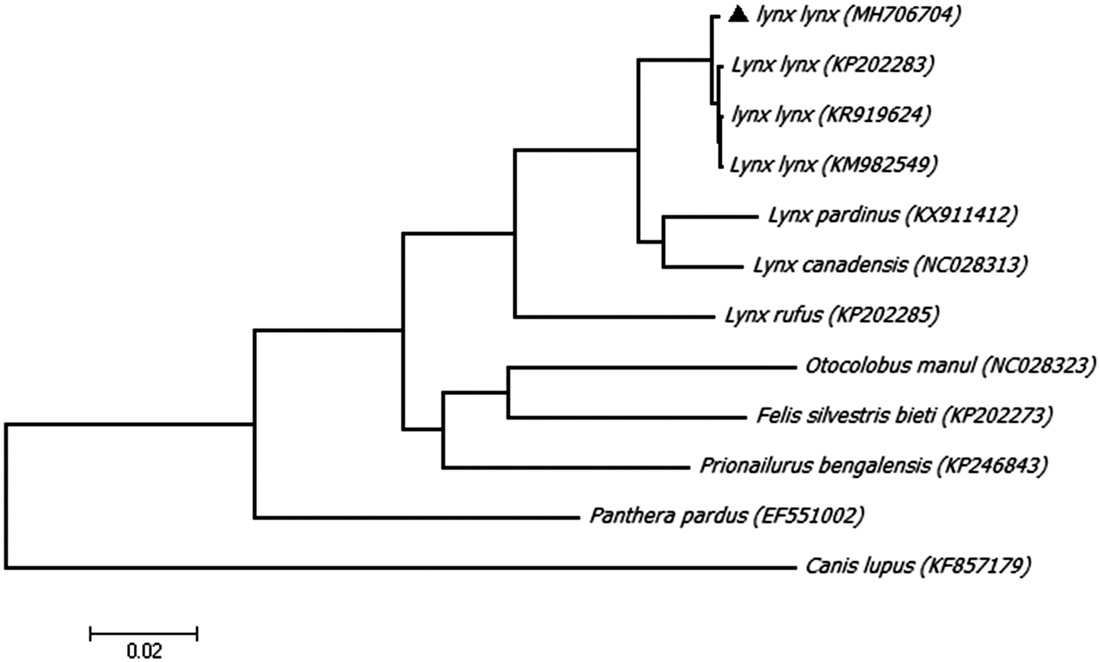
Maximunm likelihood (ML) phylogenetic tree of Eurasian lynx (*Lynx lynx*) and the other 11 sequences from the order Carnivora with a sequence of grey wolf (*Canis lupus*) as an outgroup. Number at each node indicates the bootstrap support values. GenBank accession numbers are given in the brackets after the species name. The sequence characterized in this study is annotated with a black triangle symbol.
